# Isolation and analysis of a non-protein low molecular weight thiol-mercurial adduct from human prostate lymph node cells (LNCaP)

**DOI:** 10.1042/BSR20201343

**Published:** 2020-06-18

**Authors:** Michael Gronow

**Affiliations:** Cambridge Cancer Research Fund Laboratory, 7 The Maltings, Cottenham, Cambridge CB24 8RE, U.K.

**Keywords:** LNCaP cells, low molecular weight thiols, thiol adduct chromatography and analysis

## Abstract

Thiol compounds present in human malignant prostate cells (LNCaP) were investigated after reaction with a mercurial blocking reagent. After extracting the cellular glutathione and some other low molecular weight (LMW) thiols using trichloroacetic acid the resulting the protein precipitate was extracted with buffered 8 M urea containing 2-chloromercuri-4-nitrophenol in an equimolar amount to that of the thiol present. After removing the insoluble chromatin fraction the urea soluble labeled adducts formed were chromatographed on G15 Sephadex. Three yellow coloured (*A*_410_ nm) fractions were obtained; first, the excluded protein fraction containing 16.0 ± 4.1% of the applied label followed by an intermediate fraction containing 5.9 ± 1.2%. Finally a LMW fraction emerged which contained 77.2 ± 3.7% of the total label applied and this was further analyzed by column chromatography, first on an anion exchange column and then on a PhenylSepharose 6 column to give what appeared to be a single component. LC–MS analysis of this component gave a pattern of mercuri-clusters, formed on MS ionization showing possible parent ions at 704 or 588 m/z, the former indicating that a thiol fragment of molecular weight approximately 467 could be present. No fragments with a single sulfur adduct (a 369 m/z fragment) were observed The adduct was analyzed for cysteine and other amino acids, nucleic acid bases, ribose and deoxyribose sugars, selenium and phosphorus; all were negative leading to the conclusion that a new class of unknown LMW thiol is present concealed in the protein matrices of these cells.

## Introduction

Thiols play pivotal roles in cellular metabolism and are particularly important in the maintenance of the cellular redox balance and the control of oxidative stress. In the case of cancer cells and tissues they have been shown to be important in radio-sensitization and protection, also in resistance to chemotherapeutic drugs.

Although many cellular thiol functions have been shown to be meditated via the cysteine groups found in enzyme or protein structures the main players in cellular metabolism are thought to be the mobile low molecular weight (LMW) thiols, often known as non-protein thiols (NPSH). In normal cells it is widely believed that the tripeptide glutathione is the most abundant LMW thiol present within these cells. However, it has also been reported that other reducing sulfur moieties, such as sulfides, sulfones (S°), and persulfides, play important roles in thiol metabolism (e.g. [1,2]). In addition, it has been mooted that H_2_S is a major signaling messenger molecule in cells and tissues [3]. It has also been reported that modified protein cysteine residues play an important role in redox stress signaling (e.g. [4])

Recent work on a human prostate cancer cells, a lymph node line known as LNCaP, has shown that thiols other than GSH are present in the deproteinized extract (acid soluble fraction – ASF) of these cells [5,6]. Following up this work, investigations have focused on the protein thiol content of the chromatin cellular pellet left after removal of the ASF which was found to contain 76.7% of the cellular thiol (43.2 ± 2.9 fmol/cell). After extracting with a powerful chaotropic solvent (buffered 8 M urea solution) containing excess Ellman reagent and removal of the labeled chromatin/DNA pellet it was shown by gel filtration chromatography that only 17.9% of the measured thiol could be found in the protein itself; 56.5% of the total cellular thiol appeared to be LMW material which did not give an identifiable Ellman adduct [7]. Therefore, in order to identify these thiol compounds a fresh analytical technique had to be developed.

**Figure 1:**
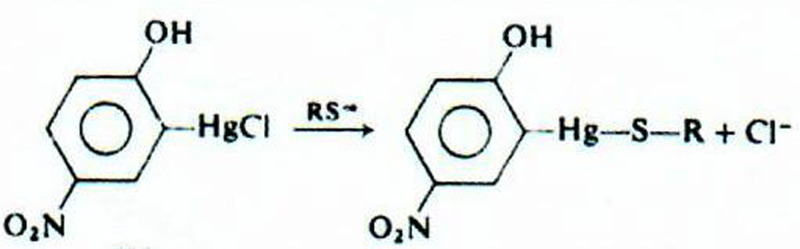
Reaction of thiols with 2-chloromercuri-4-nitrophenol

It is essential when isolating thiols for analysis that the group is blocked with a satisfactory agent to prevent oxidation artefacts. The use of most chromophoric or fluorescent thiol reagents as blocking agents is limited by their insolubility in aqueous media which can be a problem for their use in cell studies other bioanalytical systems. To this end some water soluble organo-mercurial labels offer many advantages for use in thiol analysis; they react quantitatively with thiols under appropriate conditions, are highly specific and can be easily detected using various UV/visible chromophores [8]. In the present study, 2-chloromercuri-4-nitrophenol (ClMNP) [9] has been used which reacts with thiols to give chromophoric adducts with a λ_max_ at neutral pH of 410 nm as shown below (Figure 1).

Using this reagent, the yellow adduct(s) of LMW thiols concealed in the protein matrix of human prostate cancer cells (LNCaP) were isolated and examined by various chromatographic and other analytical techniques

## Materials and methods

All reagents and chemicals were of analytical or higher grade. Ellman reagent (5,5′-dithio-bis-2-nitrobenzoic acid), Whatman D52 cellulose anion exchanger, and other chemicals used were obtained from Sigma–Aldrich (Merck) and VWR Chemicals (BDH Prolab). G15 Sephadex and PhenylSepharose 6 Fast Flow (high substitution) were supplied by GE Healthcare.

The chromophoric organomercurial ClMNP was prepared by the method of McMurray and Trentham [9]. This reagent was dissolved in acetone at a concentration of 5 μmol/ml just prior to its use in the labeling of cellular thiols in buffered 8 M urea solutions.

Glass chromatography columns were supplied by Soham Scientific, Fordham, Ely, Cambridgeshire, U.K.

LC–MS work was carried out by BioCity Group, Pennyfoot St, Nottingham NG1 1GF, U.K. This analysis was performed using a Waters Acquity H-class QDA-PDA system.

The sulfur, selenium, and phosphorus contents of adducts were determined by Inductively Coupled Plasma Mass-Spectrometry (ICP-MS) using a Thermo Finnigan Element 2 Magnetic-Sector ICP-MS via Oxford University Innovation Services by Philip Holdship at the Department of Earth Sciences, University of Oxford.

Full cysteine and other amino acid assays were carried on an ion exchange auto analyser by the Protein & Nucleic Acid chemistry facility of the Department of Biochemistry at the University of Cambridge.

LNCaP (androgen-sensitive human prostate adenocarcinoma cells, clone FGC-ECACC no. 89110211) were purchased from the Public Health England Laboratories (ECACC – HPA) at Porton Down, Salisbury, England. Cells were grown to confluence in cell factories in a medium consisting of RPMI 1640 + 2 mM glutamine + 1 mM sodium pyruvate containing 10% Zone 2 FBS. The confluent cells were harvested by trypsinization (Tryple Express) and cell counts were performed on a Nucleocounter NC3000. Aliquots containing 5 × 10^8^ cells were collected by centrifugation, snap frozen and stored at minus 80°C until required.

## Methodology employed

### Cell extraction technique – procedure used for 5 × 10^8^ cells

The cell pellet was re-suspended in water at 0–4°C to a volume of 20 ml and briefly sonicated for 1 min (2 × 30 s bursts). 2 ml of 100% TCA was added to give a final concentration of 10% w/v and well mixed, then sonicated for a further 1 min and left for 15 min in ice. The mixture was then centrifuged at 3200×***g*** for 4 min. After aspiration of the supernatant the cell residue was extracted with a further 10 ml of 10% TCA. The two TCA extracts were combined to give the ASF (approximately 32 ml) which contained the cellular glutathione.

The cell residue was then re-suspended in 20 ml of water and the thiol content determined on three to four aliquots as follows:

Triplicate samples consisting of 40 μl of cell suspension added to 2960 μl of 8 M urea 0.5 M Na phosphate buffer pH 7.6 containing 0.25 mg/ml of the Ellman reagent (5,5′-dithio-bis-2-nitrobenzoic acid – ESSE) were prepared. The *A*_412_ was read against the appropriate blank reagent and the number of optical unit/ml (in a 1 cm cell) calculated. From this value protein thiol content was ascertained by dividing by 13.7 (the millimolar extinction coefficient of the yellow anion (ES) in buffered 8 M urea solutions, see [10]).

The cell residue mixture was then centrifuged as before and the pellet re-suspended in 10 ml of water. This was then added dropwise, with rapid mixing, into 60 ml of 8 M urea 50 mM phosphate pH 7.5 (at room temperature) containing the calculated quantity of ClMNP dissolved in acetone at a 1:1 molar ratio to the thiol present. (If the cell residue suspension is added to the buffered urea without the labeling agent the thiol groups present rapidly disappear.)

After 20 min stirring, the resultant yellow mixture was then centrifuged at 3500 rpm for 8–10 min to sediment the chromatin (DNA–histone) complex [11] and the supernatant processed by gel filtration chromatography.

### Chromatographic separation of the mercuri-4-nitrophenol thiol adducts (RSMNP)

- Gel filtration on G15 Sephadex:
  2.93 g of NaCl was added to 50 ml of the supernatant (to give a 1 M NaCl concentration) and the mixture was applied to an 8 × 6 cm column of Sephadex G-15 (bead size 40–120 μm, exclusion limit 1500 Da) made up in 8 M urea 50 mM phosphate pH 7.8. The column was initially eluted in this solvent containing 1 M NaCl, collecting 10 ml fractions. The excluded protein fraction was eluted in the first 60–70 ml, after which the LMW fraction, the movement of which was retarded, was eluted in 8 M urea 10 mM ammonium bicarbonate.
  The optical density at 410 nm of the fractions [9] was determined manually in a 1 cm cuvette using a Jenway 7315 spectrophotometer.
- Anion exchange chromatography on DE52 cellulose:
  The LMW fractions obtained from the G15 column were pooled and applied to a 10 × 3 cm column of Whatman DE52 in the Cl^−^ form. After an exhaustive water wash to remove any remaining urea the yellow RSMNP was eluted in 0.2 M NaCl.
- Hydrophobic interaction chromatography:
  Further purification and removal of salts from the DE52 0.2 M NaCl fraction was achieved by applying the RSMNP to a 20 × 3 cm column of PhenylSepharose 6 in water. Up to 100 ml was applied and after a brief wash with 0.5 M NaCl the yellow adduct was eluted slowly with water. A tight yellow band is slowly formed finally eluting in about one-tenth of the sample volume applied.

### Analysis of the low molecular weight RSMNP

- Thin layer chromatography (TLC):
  Concentrated RSMNP samples were run on silica or cellulose TLC plates at neutral pH in a variety of solvents.
- Reversed phase ultra performance liquid chromatography and mass spectral analysis:
  As mercury has seven stable naturally occurring isotopes it produces a very distinctive pattern when ionized using ESI mass spectrometry, making it very easy to spot Hg adducts. Samples were analyzed on single quadrupole QDA systems in full scan mode with analyte specificity attained by using the UV λ_max_ of 323 nm of mercuri-nitrophenol ion (at acid pH) to determine peaks of interest.
  The LC column used was a charged surface hybrid (CSH) C18 2.1 × 100 mm, 1.7 μm at 50°C using the following elution program in which the mobile phases were water (A)/acetonitrile (B) both with 0.1% (v/v) formic acid. The flow rate was 0.6 ml/min:

**Table:** 

Time (min)	Initial	0.5	6.5	7.5	7.6	8.0
%A	98	98	2	2	98	98
%B	2	2	98	98	2	2
- Further analytical tests:
  Full cysteine and other amino acid assays were carried on an ion exchange auto analyser by the Protein & Nucleic Acid chemistry facility of the Department of Biochemistry at the University of Cambridge.
  The compound was checked for ribose/pentoses using the Bial’s Orcinol method and for deoxyribose (2′-deoxypentose sugars) using the Dische diphenylamine reagent.

## Results

### Thiol contents of cellular fractions

**Figure 2:**
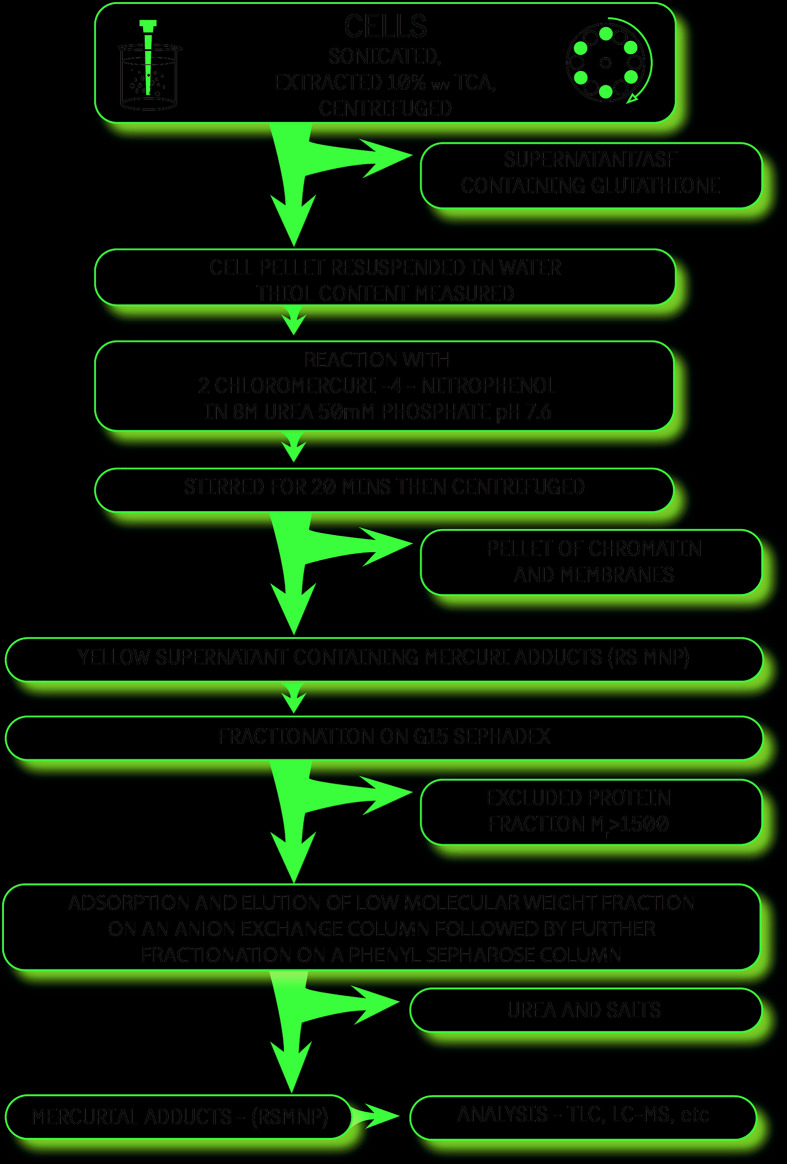
Scheme of analysis that evolved during this investigation

The thiol contents determined using the Ellman reagent on the cellular fractions obtained in the present (as shown in Figure 2) and previous studies [6,7] are given in Table 1 below. In earlier studies, the G15 protein thiol concentration was calculated from the adduct obtained from the Ellman reagent. Subtraction of the value and that obtained from the chromatin pellet gives the LMW thiol content present in the protein precipitate.

**Table 1:** Thiol contents of LNCaP cellular fractions

Thiol fraction	Femtomoles of thiol/cell	Percentage of total thiol
Total cellular thiol	56.3 ± 3.6	100.0
Glutathione	8.3 ± 0.7	14.7
Minor ASF LMW components	3.5	6.4
Protein fraction-gel filtration	11.4 ± 0.3	20.2
Chromatin pellet	1.3 ± 0.2	2.3
Protein matrix release – LMW	31.8 ± 2.2	56.4

### Gel chromatography of buffered 8 M urea extract of cells

**Figure 3:**
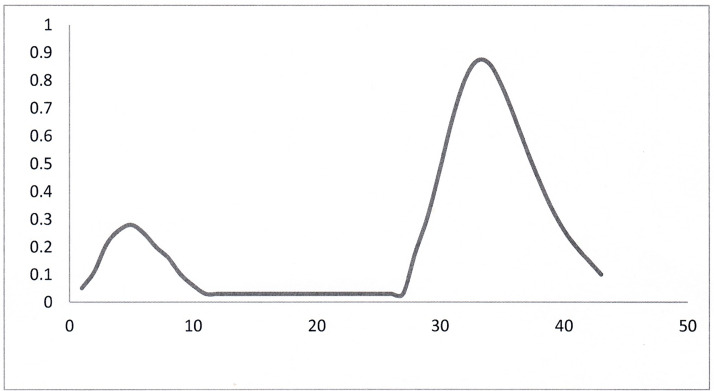
Gel filtration chromatography of cellular thiol adducts on G15 Sephadex Ordinate: OD at 410 nm; abscissa: tube no. (10 ml fractions).

Figure 3 shows the pattern obtained from chromatography of the 8 M urea extract containing the thiol adducts after the addition of NaCl to 1 M concentration on an 10 × 6 cm G15 column (made up in 8 M urea 50 mM phosphate pH 7.8). The first peak, the protein fraction, is eluted with this solvent and appears in the void volume in just over the initial volume applied. After the protein had been eluted the elution of the LMW adducts appeared to be retarded in the initial eluent. After some experimentation it was found that the best way to elute this fraction was with 8 M urea 10 mM ammonium bicarbonate after the protein had been eluted.

The amounts of *A*_410_ eluted in the G15 fractions obtained are shown in Table 2. 90+% recovery of the applied *A*_410_ was achieved.

**Table 2:** Separation of RSMNP components on Sephadex G15

Percentage of applied *A*_410_ recovered*		
Protein	“Intermediate”	LMW
16.0 ± 4.1%	5.9 ± 1.2%	77.2 ± 3.7

* Average 4 runs (±SD).

[Note that it has not been possible to accurately quantify the thiol content of the protein or the LMW fraction as the extinction coefficients of these RSMNP derivatives have yet to be determined.]

### UV-VISIBLE spectrum of low molecular weight RSMNP adduct at pH 7.5

**Figure 4:**
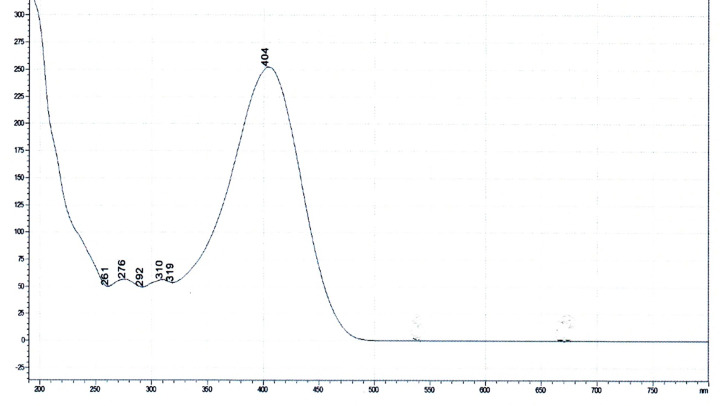
UV-VIS spectrum of LMW weight RSMNP adduct at pH 7.5 Ordinate: absorbance (mAU); abscissa wavelength (nm).

The spectrum of the RMNP isolated on a PhenylSepharose column is given in Figure 4. The major peak observed at neutral pH is at 404 nm, at acid pH this shifts to 323 nm.

### LC–MS chromatography of RSMNP

**Figure 5:**
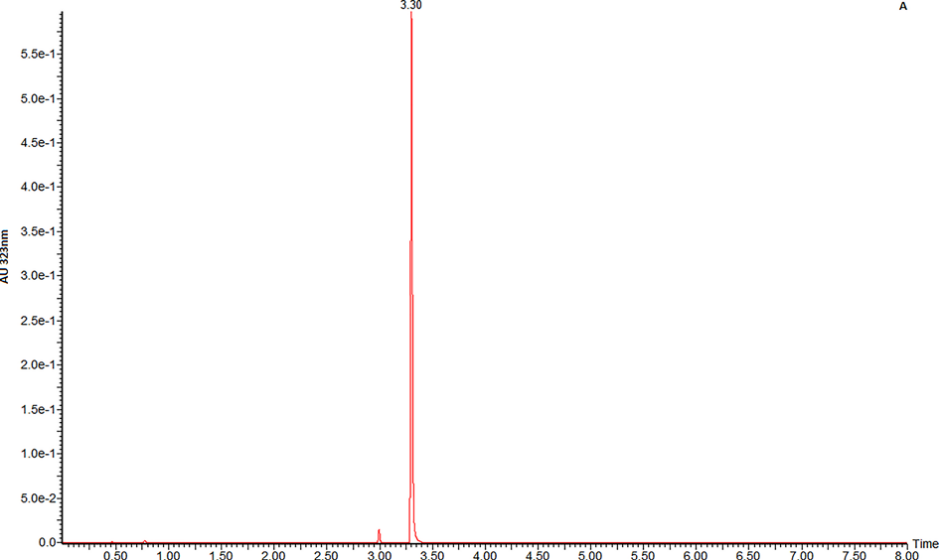
LC trace of LNCaP-RSMNP at 323 nm Ordinate: AU 323 nm; abscissa time of elution (min).

LC analysis was carried out using an acetonitrile formic acid gradient as described above in the Materials and methods section. It can be seen from the UV trace at 323 nm (λ_max_ in acid solution) in Figure 5 that only one significant component is present.

**Figure 6:**
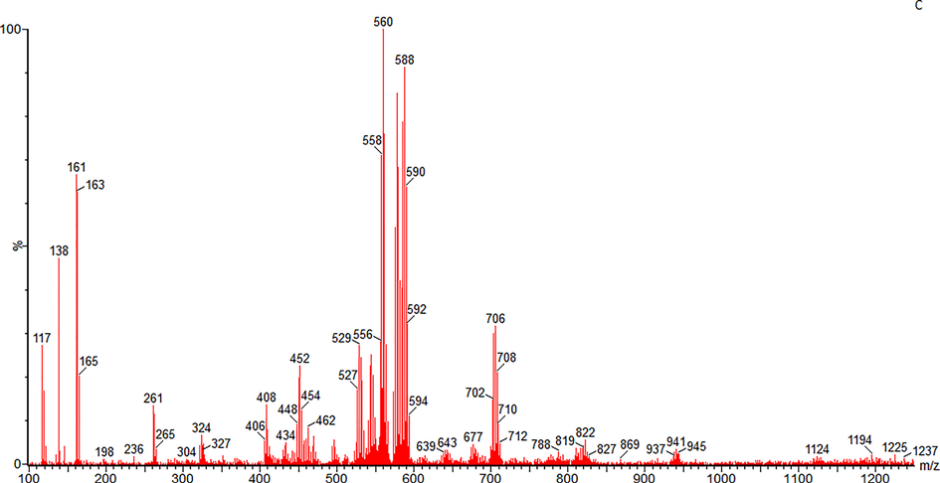
Negative ion mass spectrum of 3.3 min peak

The MS obtained from the ion trace obtained from this peak revealed a complex pattern as shown in Figure 6.

Interpretation of this MS trace is difficult as mercurial compounds often form dimers or trimers in an ionization beam. Taking the 706 m/z peak and subtracting the label mass of 338 Da (2-mercuri-4- nitrophenol minus chlorine) indicates a thiol mass of 467 could be present, if it contains only one Hg atom However, the 588 m/z peak could contain thiol with a mass of 251 assuming only one Hg is present.

The formula weight of the label 2-mercuri-4- nitrophenol plus sulfur, should be 370 Da but no ions of this size were found.

Further MS–MS analysis will be required to resolve this issue, but this data strongly indicates that this matrix thiol is not a simple sulfide, persulfide, or some form of sulfane (S°) sulfur.

The glutathione adduct was prepared and analyzed in this system. A single peak was observed which produced the expected negative ion of 645 m/z (339 + 306) (see Supplementary material).

### Other investigations

The following other investigations were carried out:

TLC investigations on silica and cellulose plates using a number of different solvents gave a single visible yellow spot (example given in Supplementary material).

The quantitative content of sulfur, selenium, and phosphorus content of the adduct was investigated by ICP-MS (linear working range from µg/g levels down to pg/g levels). While the sulfur content agreed closely with the thiol values determined by the Ellman reagent, selenium was not detected in the adduct. Only traces of phosphorus were found, for example, in one sample containing 860 nmol of sulfur only 3 nmol of phosphorus were detected indicating that the latter was only a contaminant.

Tests for cysteine and other amino acids by automated analysis were negative.

Quantitative tests for ribose/pentoses and deoxyribose (2′-deoxypentose sugars) were negative.

A low absorption in UV at 260 nm of the RSMNP adduct, allowing for that due to the nitrophenol component, indicated the absence of any nucleic acid bases.

The adduct is very soluble in water and seems to have amphoteric properties as it can easily be adsorbed onto either anion or cation exchange columns.

## Discussion and conclusion

These results obtained using 2-mercuri-4-nitrophenol to label the cellular LMW thiols confirm the findings of previous experiments on LNCaP cells using the Ellman reagent. In fact, the results presented here mirror those obtained with the latter reagent [7] in that the main bulk of the measured protein thiol can be dissociated from the cellular matrices in a strongly chaotropic solvent. Using buffered 8 M urea it has been shown that only about one-fifth of the detected thiol is firmly within the protein peptide structure, it seems as if the bulk of the cellular LMW thiol material, approximately 56–61%, is “concealed” in the protein matrices; trapped in the trichloroacetic acid precipitate.

In this context it is interesting that in previous studies by the author on the thiols of isolated **nuclei** from rat liver and rat hepatoma 223 ascites cells labeled with the Ellman reagent, similar results were obtained [12]. Here the non-histone proteins, which contain the bulk of the nuclear thiol material, were shown to form LMW thiol adducts with ^35^S labeled Ellman reagent, but these were not identified. However, it was established that they did not contain either cysteine or glutathione; furthermore only 30% of the ^35^S labeled adducts formed were found to be bound to the protein cysteine residues.

In later work by the author it was found that traces of unknown thiol moieties were detected in the ASF/glutathione fraction isolated from LNCaP and other cells [5,13].

The present study shows that the reducing sulfur entity entrapped in the protein matrices of LNCaP cells is not a simple divalent sulfur moiety and does not contain the amino acids, cysteine, or glutathione.

The amount of the unknown thiol present in LNCaP cells is approximately four times greater than the glutathione present indicating that it must play an important role in the control of oxidative stress and other well-known thiol functions. At a concentration of 31.8 fmol/cell it could represent an important element in energy metabolism, in contrast, for example, the amount of ATP present in a cell is only 1–2 fmol [14].

This concealed or “conthiol” reducing sulfur entity is not any of the known cellular constituents. In order to identify it fully new analytical methods need to be developed.

Elucidating the role of these “conthiols” in oxidative stress, hypoxia, redox regulation, signaling, and cellular respiratory processes poses a major challenge in future research programs and studies. However, from the current knowledge of thiol metabolism it is evident that they must be important in relation to tumour cell response to ionizing radiation, drug resistance, and oxidative stress generally. Elucidation of their chemistry and biochemistry would seem to hold some promise for helping to improve the design of more effective radiosensitizers and cancer chemotherapeutic drugs.

## Supplementary Material

Supplementary Material

## Abbreviations

ASF: acid soluble fraction
ClMNP: 2-chloromercuri-4-nitrophenol
ICP-MS: inductively coupled plasma mass-spectrometer
LC–MS: liquid chromatography-mass spectrographic analysis
LMW: low molecular weight
LNCaP: lymph node androgen-sensitive human prostate adenocarcinoma cells (clone)
RSMNP: mercuri-4-nitrophenol thiol adduct
TLC: thin layer chromatography
