# Supplementary material for: Isolation and analysis of a non-protein low molecular weight thiol-mercurial adduct from human prostate lymph node cells (LNCaP)
Source: Biosci Rep. 2020 Jun 18;40(6):BSR20201343. doi: 10.1042/BSR20201343 (PMC7303348; doi:10.1042/BSR20201343)

SUPPLEMENTARY MATERIAL

1) THIN LAYER CHROMATOGRAPHY OF THE ADDUCT

TLC analysis of the 2-mercuri-4-nitrophenol adducts on various silica, RP C18 or cellulose plates at neutral pH showed only one major yellow component to be present.

An example of a TLC separation on a silica plate (Al): solvent 30% EtOH, is shown below.

TLC plate of labelling compound ClMNP (LHS) and adduct RSMNP (RHS)

2) GLUTATHIONE ADDUCT

LC analysis of glutathione -2 mercuri-4-nitrophenol adduct

a) LC-MS - UV trace

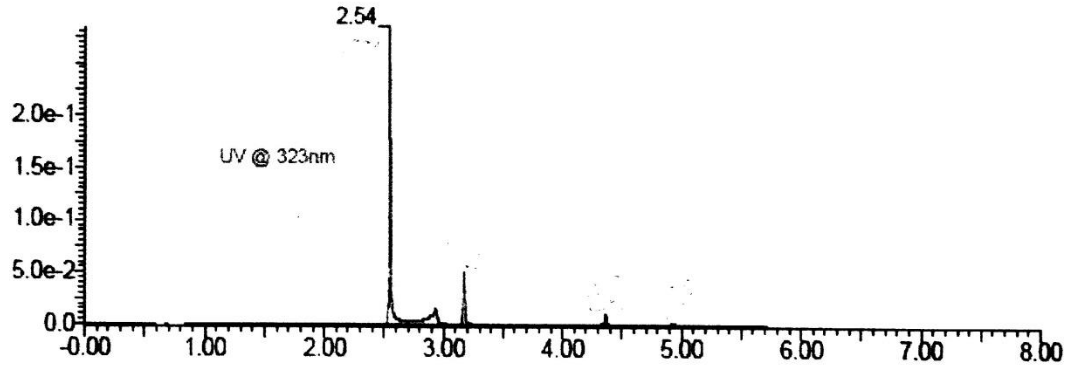

b) Negative ion MS analysis of above 323nm peak eluting at 2.54 mins

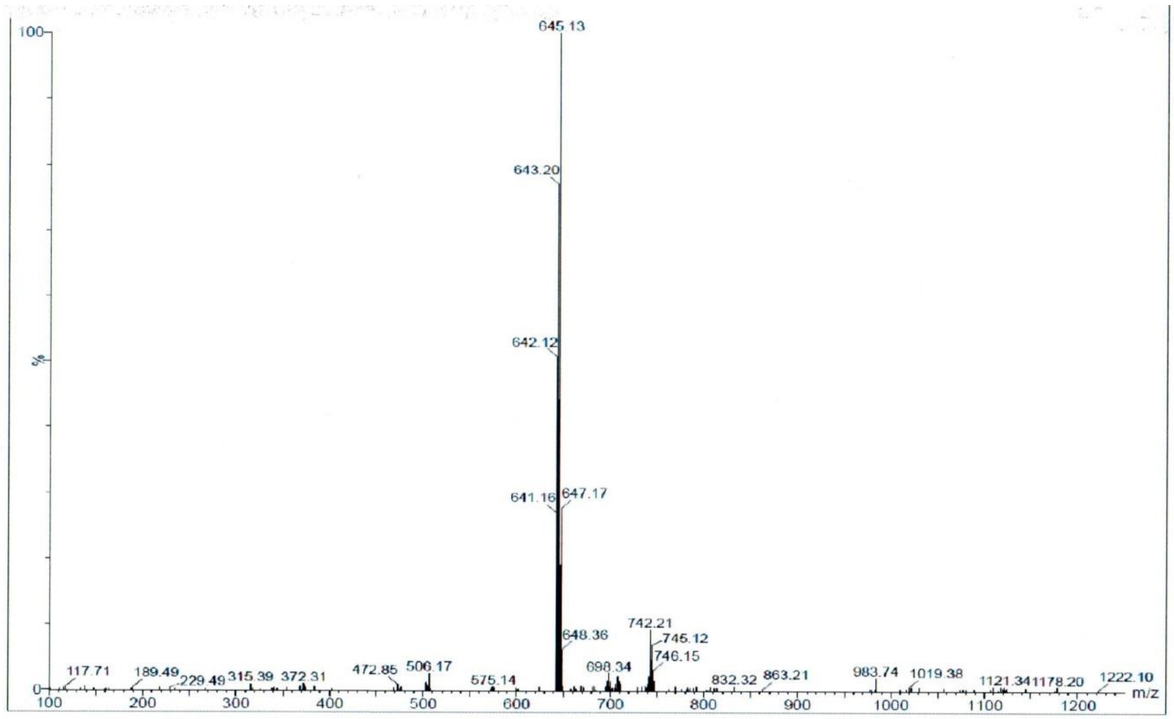

Supplement: Supplementary Material [file BSR-2020-1343_supp.pdf]
